# News and Miscellany

**Published:** 1883-01

**Authors:** 


### NEWS AND MISCELLANY.

TSETSE FLY.—Man, the goat, and the wild carnivora are the only animals capable of resisting the tsetse fly of Africa.

OSTRICH BREEDING.—One good pair of breeding birds, well fed, hatches out a clutch of chickens three or four times in the year, from twelve to twenty chickens in each clutch. All breeding birds are not of course equally good, some will perhaps not produce more than half this number of chickens, but generally such difference arises from their youth, or insufficient feeding; whatever the cause, these birds are always sold for a proportionately lower price.

The period required to incubate the eggs is forty-two days. A few days after one clutch is hatched, they again pair, and the female speedily lays the first egg, continuing to lay every other day until the number is complete; they then commence sitting, the hen occupying the nest, which is merely a hollow scraped out of the ground, from three or four o'clock in the morning, until the same time in the afternoon, when she is relieved by the cock, who takes charge during the night. Should the soil in the camp not be of a sandy nature, it is as well then to place a cartload of sand there, as it makes a better nest than ordinary soil, being more porous and drier.

RATION OF HAY FOR HORSE.—The experiments of Wolff and others, at German experiment stations, show that a horse weighing 1100 to 1200 lbs., would eat from 22 to 27½ lbs. of hay, if no other food was given. With grain, 20 to 25 lbs. was usually eaten by working horses of that weight. Lighter horses would not need quite so much, but we can find no data of experiments with horses weighing less than 1000 lbs.—*Country Gentlemen.*

THE DAILY YIELD OF GOATS' MILK.—The yield of milk from a goat averages about two to three pints per day for seven or eight months in the year, and she gives three or four kids, in two kiddings, to be killed at three to six weeks old, a delicate, choice meat, superior to lamb.

DONATION TO VETERINARY COLLEGE.—A subscription of $10,000 from Mr. J. B. Lippincott, for the establishment of a veterinary college in connection with the University of Pennsylvania was received by the Trustees of that institution, November 14, 1882.

THE BERLIN VETERINARY MEDICAL SCHOOLS.—The Berlin schools cost more than $20,000 per year above receipts, with large faculties. Berlin has thirteen professors and a large number of assistants, and is erecting institutes for the study of special branches; the new pathological institute at Berlin is to cost $50,000; at least such is the appropriation, It is from the work done at these schools that we have learned how to control animal pests, and the nature of most of the diseases of our animals.—*F. S. Billings.*

NORTH-WESTERN VETERINARY COLLEGE.—The prospectus of the second session of this Institution has been received. Dr, Lyford the Principal has wisely insisted from the outset on a high standard, and requires each candidate for the diploma to pass an entrance examination and to spend three winter sessions and one summer session. The College is connected with the Minnesota Medical School, and doubtless the association will be mutually beneficial. Dr. Lyford is an alumnus of McGill University.

PHILADELPHIA VETERINARIANS AND DISHONEST DRUGGISTS.—It seems that the "City of Brotherly Love" still harbors some dishonest compounders of drugs, and that the *Press* has undertaken to rebuke the profession for the short-commings of its unworthy members. A certain C. B. Henry, of 1537 Bertram Street, prescribed the following:

"FOR MR. BYERLY'S DOG.

℞ Quinine Sulph........................1 drachm.
Acid Sulph. dil........................q. s.
Aqua........................4 drachms

Mix. 20 drops as directed."

The prescription was sent to apothecary F. Pleibel, who, it is asserted, substituted the cheaper sulphate of cinchonidia for the sulphate of quinine ordered. Whether "Mr. Byerly's Dog" first discovered the cheat, or whether some employe of the *Press* assumed the role of public guardian, is not stated.

In like manner the same C. B. Henry, V. S., prescribed two drachms of Boracic Acid, and druggist Charles H. Gmelin of Third and Poplar Streets substituted Borax therefor.—*Sanitary Engineer.*

BLOODPOISONING.—Our animal-trainers are apt to be careful hereafter how, in seeking to save the cost of pumice-stone soap or sand-paper, they wash their faces by rubbing them against the hide of an elephant. Professor Arstingstall nestled his countenance against the trunk of the elephant Chief in Bridgeport, Ct., one day last week, and now his skin shows an eruption that physicians regard as a bad case of poisoning.—*Clipper.*

POISONING BY ARSENIC.—From experiments on eight dogs. Dr. E. Ludwig concludes that in all cases, whether of acute or chronic poisoning, the liver is the organ richest in arsenic. The liver was found to contain nearly seventeen times as much of the poison as the brain. To this the observations on men poisoned by arsenic lent strength. In a typical case of acute poisoning the brain contained 100 grammes, 0,00004 gramme; the liver, per 100 grammes, 0. 00338 gramme of arsenic. It would, therefore appear that the liver, is the best part to examine in judicial cases.

A CAT'S ATTEMPT AT SUICIDE.—A strange incident occured on the dock adjoining the Staten Island ferry slip. An old cat belonging to Mr. Stephens, the Secretary of the Staten Island Ferry Company, was observed standing at the edge of the dock, when she suddenly plunged into the water. A Police officer went to the rescue of the cat and landed it safely. The officer believes that the cat intended to drown itself. It is twelve years old, and has of late been quite infirm, being partially paralyzed. After it had been rescued the cat was looked up in a safe place, where it will have no facilities for making another rash attempt upon its own life.

WHAT IS CRUELTY TO A CAT.—The trial of Hugh Devlin for cruelty to a cat called forth interesting medical testimony at Providence on Tuesday. Devlin had confessed that, having been annoyed by a huge tom-cat, he had chucked it alive under the ground, not wantonly, but because he thought that the surest and most merciful way to take the animal's nine lives. The agent of the Humane Society contended that Devlin had done a cruel, wanton deed, but physicians testified for the defence that death by suffocation under ground was for any animal as easy and painless as death by drowning. The popular horror of being buried alive had its origin, not in experience, but in the imagintion, which pictured such a fate as terrible. Whatever Devlin's intent, he had caused the cat no more death agony than if he had followed the orthodox method and drowned it. Possibly, said one physician, the cat lived longer by three respirations than it would have done under water. Devlin was found not guilty of cruelty to a cat.—*Springfield Republican.*

OVERFED PIGS.—When young pigs are sick it may be pretty certainly understood that they have been overfed. The general treatment of pigs seemed to be based upon the idea that they are naturally greedy and gluttonous animals, and that this habit should be encouraged as much as possible. Hence all the diseases which so frequently effect pigs. When young, a pig is a tender animal, with a stomach not much larger than that of an infant about as old, and yet the people will cram the little creature with sour slop, grease, milk, and corn-meal until it can swallow no more. And when the pig is sick one wonders what is the matter. We do not feed lambs or calves, or colts, in that fashion, hence these are rarely diseased. Cough and difficulty of breathing is caused by indigestion, and the common disease of which partial paralysis of the hind parts is the chief sympton, and which is cerebro-spinal meningitis, is caused by indigestion and malnutrition, which cause disturbance of the circulation and congestion on the brain and spinal marrow, with loss of nervous power. The treatment is to give a dose of salts and one scruple of saltpeter daily afterward, and feed very sparingly.—*Dublin Farmers' Gazette.*

"A Cow as is a Cow."—Hon. Harrison Bailey owns a cow which, in the last four years, has dropped nine calves, three of them the present year, and of course is highly valued. A few nights ago she got into a cornfield and filled herself so full of the provender there that she was unable to rise when she laid down, and nothing short of her death was expected until a neighbor came along, and undertook to relieve her by plunging a butcher-knife into her flank just in front of the hip bone. This failed to have the desired effect, and another neighbor proposed to enlarge the hole made by the knife and remove the food. To this the owner would not consent until convinced that she would die any way, and then the experiment was tried, and fully six bushels of undigested corn, cornstalks and grass, in a state of fermentation, was removed from her paunch. The opening was then closed, and in a very short time the animal was on her feet and as contentedly chewing her cud as if nothing unusual had happened, and is to-day as well as any other cow on the place.—*Shelbyville (Ky.) Sentinel.*

Parasites In Stock.—In the spring, when the enemies are more or less troublesome, take common bar or soft soap, heat with a little water till melted, then add carbolic acid crystals in the proportion of one ounce to each pound of soap. Before adding the crystals to the soap, they are to be dissolved by removing the cork and setting the bottle in warm water. When the mixture is cool, make a strong suds by mixing in a pailful of warm water about 1½ lb. of the preparation; wash the infected animals with the suds. If the first application does not effect a cure try a second, and a third, with five days' intervals, It will not take off the hair, but it will take off all insects and will cure mange, or other skin diseases.

International Veterinary Congress.—Mr. Fleming, Army Veterinary Inspector, has been appointed by the Organizing Committee of the International Congress, which meets at Brussels next year, to prepare a report on association with Mr. Lydten, Superior Veterinary Surgeon to the Grand Duchy of Baden, on tuberculosis, from a pathological and sanitary point of view; the report is to serve as a basis for the discussion which is to take place on this malady.—*London Veterinary Journal.*

Cattle Quarantine Stations.—With regard to the selection of sites for quarantining imported cattle for ninty days from date of shipment in Europe, we note the following statements:

At Portland, Me., grounds have already been selected, and buildings thereon are in readiness to receive a limited number of cattle, but the government has not as yet begun the erection of its stables. Near Baltimore a place close to the intersection of the Baltimore & Ohio and the Baltimore & Potomac railroads, nine miles from the city, has been chosen. About twenty acres on high and well drained ground will probably be inclosed and buildings put up as soon as possible after the necessary formalities are gone through with.

The Commission has notified Collector Robinson of New York, that it has selected a site at Paterson, N. J., for the establishment of a cattle quarantine station. The ground that has been chosen can be completely isolated at will. One of the chief reasons which led to its selection is the fact that it can be reached either by land or by water. Many importers would prefer to have their cattle transported to quarantine by water, as [illegible]

stock may often be injured by being carried in the cars. The United States government owns land at Sandy Hook which could be used as a quarantine without the payment of ground rent. Fresh water can be had there for cattle by sinking wells twelve feet deep. The Commissioners decided against Sandy Hook because of its distance from the city. The cattle would have to be taken from the steamship in barges to the Hook, and this would be difficult in rough weather, for the barges would be in danger of swamping.

It is not likely that a station will be immediately established at Philadelphia, but it is not unlikely that the interests of the South will be considered, and a station in New-Orleans be built. At each port where cattle from abroad may be landed the collector of customs will take charge of the stock, and it will remain under his care until the agent of the cattle commission shall have provided clean box cars for its transportation from the dock to the quarantine station. The commission will then receive the stock, and, without allowing it to come in contact with any other cattle, or with material of any kind which can have by any means become infected, will take it to the quarantine grounds, where it will remain for the prescribed period under the constant supervision of veterinarians of approved skill.

The government will furnish only shelter and water, the owners will have to provide for the care and feeding of the cattle.

Exports and Importers and Quarantine of Neat Cattle.—The number of live cattle exported, chiefly to Great Britain for the year ending June 30, 1881, was 185, 707, valued at $14,304,103. For the year ended June 30, 1882, the number was 108,110, a decrease of 77,597, and in value of $6,503,876. The decrease was, however, no greater in proportion than that generally in the exportation of articles of food. By an order of the Privy Council of Great Britain of Febuary, 1879, all cattle imported from the United States must be slaughtered at the port of arrival within ten days. This order, deemed necessary to prevent infection, will, no doubt, be rescinded whenever the United States adopt measures rendering it reasonably certain that importations of cattle from this country will not introduce the disease from which the people of Great Britain have heretofore suffered loss. Under a recent appropriation the Treasury Department, through the Cattle Commission, are arranging with the various railroad companies for the transportation of cattle from the Western States to the sea-board so as to save them from contagion on the route. When these arrangements are perfected and found efficient we may fairly ask of Great Britain that the order for immediate slaughter of American cattle imported into that country be rescinded. The report of the commission speaks in detail on the subject.

Legislation Regarding Neat Cattle.—" Legislation on these subjects," says the Secretary of the Treasury in this report, has two objects: First, the extinction and prevention in the United States of the disease known as pleuro-pneumonia or lung plague; second; the increase of our commerce in neat cattle with other nations, especially Great Britain. The disease did not begin in this country, the first cases having been traced to foreign origin. It is found on the Atlantic coast in several places from New York to Baltimore, but has not been felt in New England for many years. It is a contagious disease, of malignant type, likely to spread through herd to herd. Mindful that the number of neat cattle in the United States in 1880 was about 36,000,000, which, at $25 per head, would be valued at $900,000,000, and that there has probably been increase rather than than decrease, it is seen that this is a matter of moment. The spread of the disease on the Atlantic coast alone would make serious loss, though it is more readily controlled where cattle are penned or housed. The starting and spread of it in the great open cattle ranches of the West would be calamitous"

Typhoid Fever among Live Stock.—Considerable excitement a short time ago created among the owners of cattle in North Hudson County, N. J., by the death of several cows belonging to A. Lieberman. The latter is a dairyman who owned ninety milch cows. Disease broke out in the herd on

Thursday, and twenty-two of them have since died. Two horses that were similarly affected have also died, and three others are hopelessly sick. Mr. Lieberman's first suspicion was that some malicious neighbor had poisoned them, but a post mortem examination of one of the carcases made by a veterinary surgeon named Chambon showed that death resulted from typhoid fever. Dr. Saltonstahl, the County Health Inspector, when applied to for information concerning the matter, refused to give any, for the reason, probably, that he knew nothing about it. Dr. Benjamin, the Health Inspector of Jersey City, took active steps to prevent the sale of the milk from he dairy in Jersey City. Mr. Lieberman pastured his cattle on the Vreeland farm, which borders on Penn Horn Creek.

SPECIMEN VETERINARIAN.—*What might be a Chapter from Dickens.* Denis O'Neil bought a horse from John H. Whitson & Son, 210 East Twenty-fourth Street, New York, for $160. The horse died and in the Circuit Court, Part I., this morning, O'Neil sued to recover $200 damages.

Plaintifl said that the defendants represented the horse as sound, kind and true, and that on that representation he bought the horse. On getting him home to Brooklyn, he would not eat and could not work, though he continued to look well. Plaintiff took the horse back and asked Mr. Whitson to exchange. Mr. Whitson said that, if they exchanged it, they would give him another one as bad, and told him to take horse back and doctor him. The plaintiff called a veterinary surgeon and the horse died.

In cross examination the plaintiff said the horse walked naturally and he liked him as well as when he bought him. When he took the horse back to Mr. Whitson, he looked at him and said: "He is sick." The blacksmith who shod him said that sometimes, when a horse changed his stable, he went off his feed for a time, and that was the reason why the plaintiff did not return him sooner.

Mr. John Skevington, of 1,820 Fulton Street, was examined by the counsel for the plaintiff.

Q. What is your profession? A. I am a veterinary surgeon, have been all my days, which I am fifty-five—least ways 25 years of it, and born in it, so to say, from childhood.

Q. What kind of a horse was this? A. Generally a very useful looking horse to look at, somewheres about eight years and fifteen hands and a quarter which is a hinch.

Q. What was the color of the horse? A. A kind of dapple gray—not white or gray—a mixed color, anywise say gray.

Q. When you examined the horse what did you find? A. No. sooner does I see him as I open the gate, leastways a barn, it was, I knew. I goes and I looks and I searches him, and when I opens his mouth it comes down and all through the system—ulcerated lungs. Call it what you like.

Q. Was the horse suffering from a long continued sickness? A. I should say by my own practise that the discharge was a considerable time. The flesh was flabby and nice and so beautiful. What they feed on most when it's this sickness is mostly nothing but their own corruption. Its a cur'ous case to me.

Q, How long had the horse been sick—three or four days? A. Three or four days! Three or four months, might be two years.

Q. How many visits did you pay the horse? A. Three and three is six—eight.

Q. What did you charge? A. Two dollars a visit, but being a industrious gentleman as works hard, I lumped the whole—$10.

By the defense—How old was the horse? A. Somewheres from eight to nine years in my judgment. He stood fifteen 'ands one hinch, a very useful looking animal, very nice. Just such a 'orse as I should choose, but, by Jingo! I never smelt sich.

Q. What did you do to discover the horse's condition? I guided him all over, searched him to see what was there. He was weak as a cat, but fresh as a mole.

Q. Would a horse in such a condition refuse food? A. Sometimes he'll heat hall he can get, and sometimes nothing you can give him. They live on their own corruption and get fat as (say) a pig itself.

By the defense—Could a horse in such a condition be driven? A. He might go a little way but it comes down green and a pinkish color in the nostrils.

Q. In such a condition, was a horse likely to be hearty and sleek in appearance? A. He couldn't be 'earty, but he'd look nice, so nice you'd like him yourself.

By the plaintiff—Can you tell whether the horse had been doctored? A. He had been drugged, for whot can't say, but somethink o'that.

Michael Ford, a stalwart blacksmith, went with the plaintiff to buy the horse.

Q. What did you think of the horse? A. I know nothing about a horse's insides, but about a horse's foot, I was brought up on that and knows what it is.

As the action was improperly brought, the court allowed the plaintiff to withdraw a juror and go to Special Term to amend his complaint.—*Brooklyn Eagle.*

